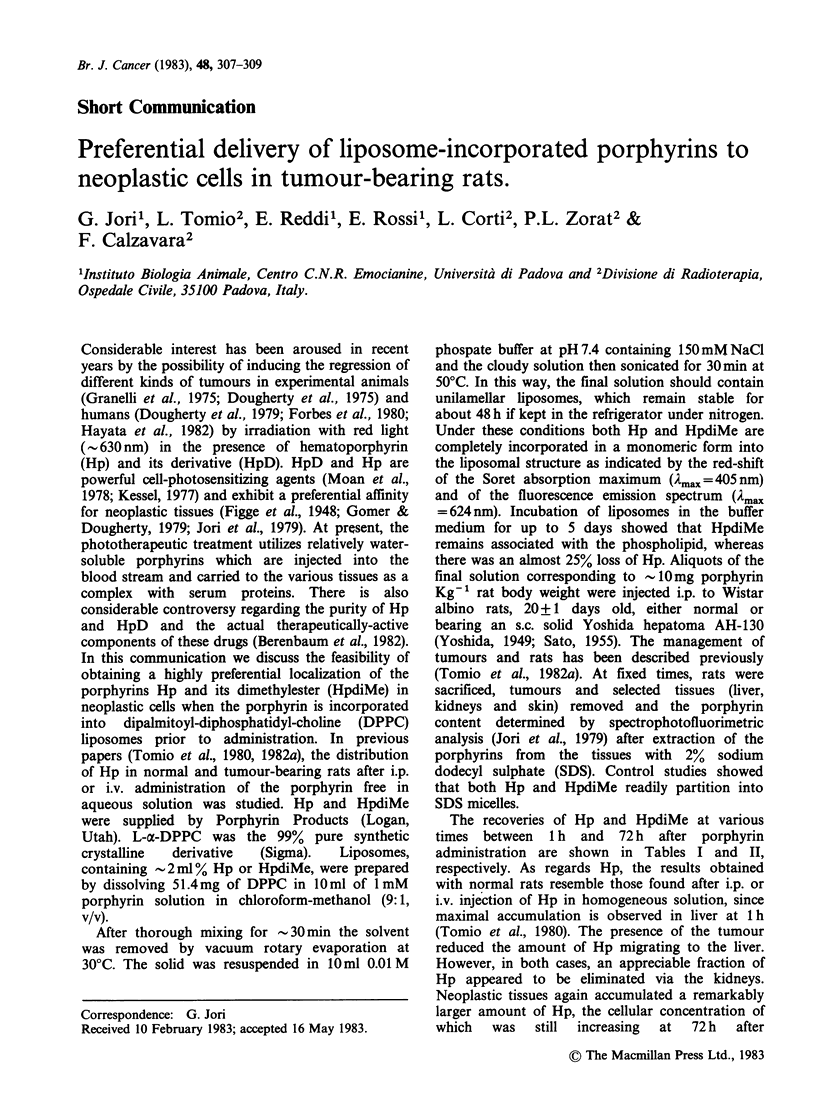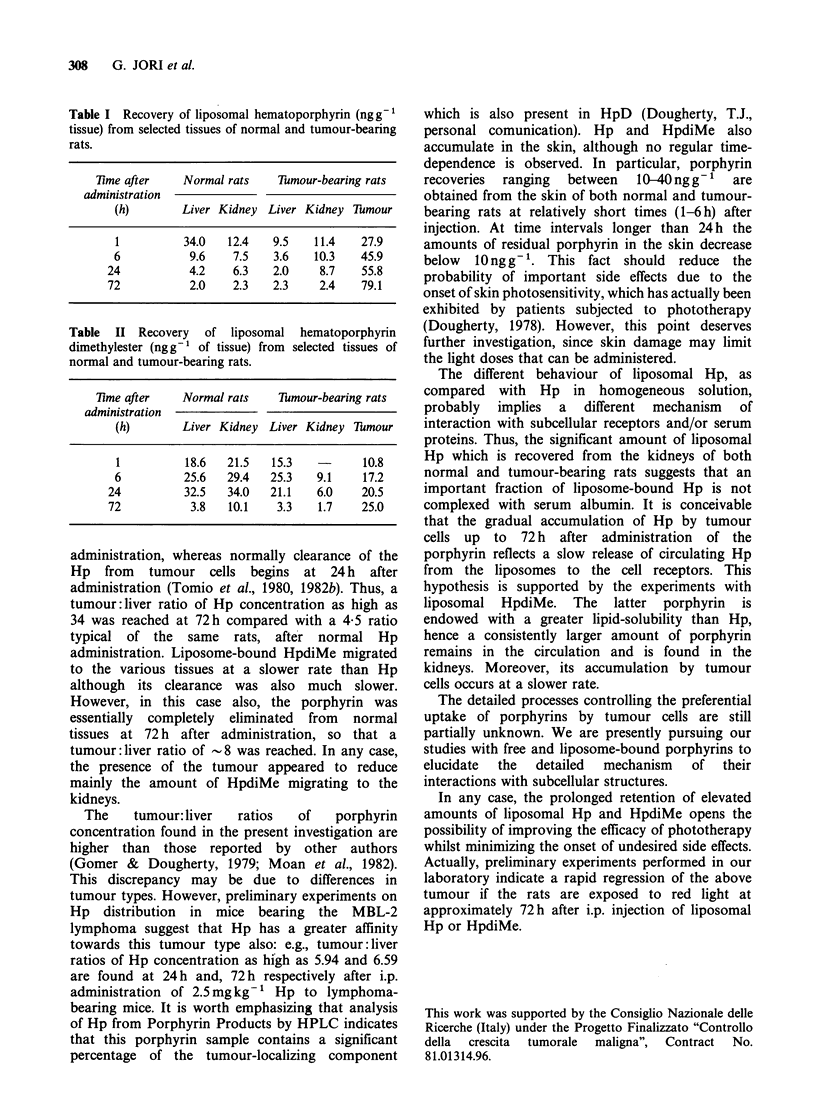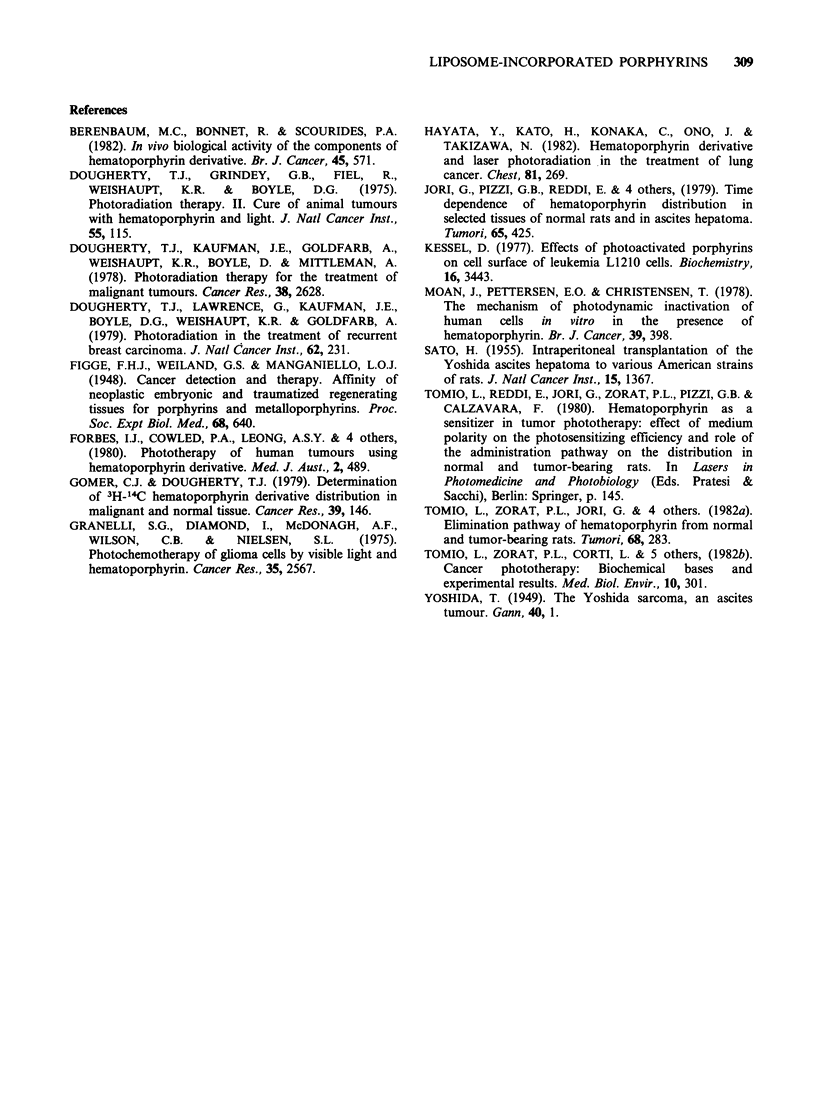# Preferential delivery of liposome-incorporated porphyrins to neoplastic cells in tumour-bearing rats.

**DOI:** 10.1038/bjc.1983.186

**Published:** 1983-08

**Authors:** G. Jori, L. Tomio, E. Reddi, E. Rossi, L. Corti, P. L. Zorat, F. Calzavara


Br. J. Cancer (1983), 48, 307–309

### Short Communication

### Preferential delivery of liposome-incorporated porphyrins to neoplastic cells in tumour-bearing rats.

G. Jori[1], L. Tomio[2], E. Reddi[1], E. Rossi[1], L. Corti[2], P.L. Zorat[2] & F. Calzavara[2]

[1]*Instituto Biologia Animale, Centro C.N.R. Emocianine, Università di Padova and* [2]*Divisione di Radioterapia, Ospedale Civile, 35100 Padova, Italy.*

Considerable interest has been aroused in recent years by the possibility of inducing the regression of different kinds of tumours in experimental animals (Granelli *et al.*, 1975; Dougherty *et al.*, 1975) and humans (Dougherty *et al.*, 1979; Forbes *et al.*, 1980; Hayata *et al.*, 1982) by irradiation with red light (~630 nm) in the presence of hematoporphyrin (Hp) and its derivative (HpD). HpD and Hp are powerful cell-photosensitizing agents (Moan *et al.*, 1978; Kessel, 1977) and exhibit a preferential affinity for neoplastic tissues (Figge *et al.*, 1948; Gomer & Dougherty, 1979; Jori *et al.*, 1979). At present, the phototherapeutic treatment utilizes relatively water-soluble porphyrins which are injected into the blood stream and carried to the various tissues as a complex with serum proteins. There is also considerable controversy regarding the purity of Hp and HpD and the actual therapeutically-active components of these drugs (Berenbaum *et al.*, 1982). In this communication we discuss the feasibility of obtaining a highly preferential localization of the porphyrins Hp and its dimethylester (HpdiMe) in neoplastic cells when the porphyrin is incorporated into dipalmitoyl-diphosphatidyl-choline (DPPC) liposomes prior to administration. In previous papers (Tomio *et al.*, 1980, 1982*a*), the distribution of Hp in normal and tumour-bearing rats after i.p. or i.v. administration of the porphyrin free in aqueous solution was studied. Hp and HpdiMe were supplied by Porphyrin Products (Logan, Utah). L-α-DPPC was the 99% pure synthetic crystalline derivative (Sigma). Liposomes, containing ~2 ml% Hp or HpdiMe, were prepared by dissolving 51.4 mg of DPPC in 10 ml of 1 mM porphyrin solution in chloroform-methanol (9:1, v/v).

After thorough mixing for ~30 min the solvent was removed by vacuum rotary evaporation at 30°C. The solid was resuspended in 10 ml 0.01 M phospate buffer at pH 7.4 containing 150 mM NaCl and the cloudy solution then sonicated for 30 min at 50°C. In this way, the final solution should contain unilamellar liposomes, which remain stable for about 48 h if kept in the refrigerator under nitrogen. Under these conditions both Hp and HpdiMe are completely incorporated in a monomeric form into the liposomal structure as indicated by the red-shift of the Soret absorption maximum ($\lambda_{max} = 405$ nm) and of the fluorescence emission spectrum ($\lambda_{max} = 624$ nm). Incubation of liposomes in the buffer medium for up to 5 days showed that HpdiMe remains associated with the phospholipid, whereas there was an almost 25% loss of Hp. Aliquots of the final solution corresponding to ~10 mg porphyrin $Kg^{-1}$ rat body weight were injected i.p. to Wistar albino rats, $20 \pm 1$ days old, either normal or bearing an s.c. solid Yoshida hepatoma AH-130 (Yoshida, 1949; Sato, 1955). The management of tumours and rats has been described previously (Tomio *et al.*, 1982*a*). At fixed times, rats were sacrificed, tumours and selected tissues (liver, kidneys and skin) removed and the porphyrin content determined by spectrophotofluorimetric analysis (Jori *et al.*, 1979) after extraction of the porphyrins from the tissues with 2% sodium dodecyl sulphate (SDS). Control studies showed that both Hp and HpdiMe readily partition into SDS micelles.

The recoveries of Hp and HpdiMe at various times between 1 h and 72 h after porphyrin administration are shown in Tables I and II, respectively. As regards Hp, the results obtained with normal rats resemble those found after i.p. or i.v. injection of Hp in homogeneous solution, since maximal accumulation is observed in liver at 1 h (Tomio *et al.*, 1980). The presence of the tumour reduced the amount of Hp migrating to the liver. However, in both cases, an appreciable fraction of Hp appeared to be eliminated via the kidneys. Neoplastic tissues again accumulated a remarkably larger amount of Hp, the cellular concentration of which was still increasing at 72 h after

Correspondence: G. Jori
Received 10 February 1983; accepted 16 May 1983.

© The Macmillan Press Ltd., 1983

**Table I** Recovery of liposomal hematoporphyrin (ng $g^{-1}$ tissue) from selected tissues of normal and tumour-bearing rats.

| *Time after administration (h)* | *Normal rats* | | *Tumour-bearing rats* | | |
|---|---|---|---|---|---|
| | *Liver* | *Kidney* | *Liver* | *Kidney* | *Tumour* |
| 1 | 34.0 | 12.4 | 9.5 | 11.4 | 27.9 |
| 6 | 9.6 | 7.5 | 3.6 | 10.3 | 45.9 |
| 24 | 4.2 | 6.3 | 2.0 | 8.7 | 55.8 |
| 72 | 2.0 | 2.3 | 2.3 | 2.4 | 79.1 |

**Table II** Recovery of liposomal hematoporphyrin dimethylester (ng $g^{-1}$ of tissue) from selected tissues of normal and tumour-bearing rats.

| *Time after administration (h)* | *Normal rats* | | *Tumour-bearing rats* | | |
|---|---|---|---|---|---|
| | *Liver* | *Kidney* | *Liver* | *Kidney* | *Tumour* |
| 1 | 18.6 | 21.5 | 15.3 | — | 10.8 |
| 6 | 25.6 | 29.4 | 25.3 | 9.1 | 17.2 |
| 24 | 32.5 | 34.0 | 21.1 | 6.0 | 20.5 |
| 72 | 3.8 | 10.1 | 3.3 | 1.7 | 25.0 |

administration, whereas normally clearance of the Hp from tumour cells begins at 24 h after administration (Tomio *et al.*, 1980, 1982*b*). Thus, a tumour:liver ratio of Hp concentration as high as 34 was reached at 72 h compared with a 4·5 ratio typical of the same rats, after normal Hp administration. Liposome-bound HpdiMe migrated to the various tissues at a slower rate than Hp although its clearance was also much slower. However, in this case also, the porphyrin was essentially completely eliminated from normal tissues at 72 h after administration, so that a tumour:liver ratio of ~8 was reached. In any case, the presence of the tumour appeared to reduce mainly the amount of HpdiMe migrating to the kidneys.

The tumour:liver ratios of porphyrin concentration found in the present investigation are higher than those reported by other authors (Gomer & Dougherty, 1979; Moan *et al.*, 1982). This discrepancy may be due to differences in tumour types. However, preliminary experiments on Hp distribution in mice bearing the MBL-2 lymphoma suggest that Hp has a greater affinity towards this tumour type also: e.g., tumour:liver ratios of Hp concentration as high as 5.94 and 6.59 are found at 24 h and, 72 h respectively after i.p. administration of 2.5 mg $kg^{-1}$ Hp to lymphoma-bearing mice. It is worth emphasizing that analysis of Hp from Porphyrin Products by HPLC indicates that this porphyrin sample contains a significant percentage of the tumour-localizing component which is also present in HpD (Dougherty, T.J., personal comunication). Hp and HpdiMe also accumulate in the skin, although no regular time-dependence is observed. In particular, porphyrin recoveries ranging between 10–40 ng $g^{-1}$ are obtained from the skin of both normal and tumour-bearing rats at relatively short times (1–6 h) after injection. At time intervals longer than 24 h the amounts of residual porphyrin in the skin decrease below 10 ng $g^{-1}$. This fact should reduce the probability of important side effects due to the onset of skin photosensitivity, which has actually been exhibited by patients subjected to phototherapy (Dougherty, 1978). However, this point deserves further investigation, since skin damage may limit the light doses that can be administered.

The different behaviour of liposomal Hp, as compared with Hp in homogeneous solution, probably implies a different mechanism of interaction with subcellular receptors and/or serum proteins. Thus, the significant amount of liposomal Hp which is recovered from the kidneys of both normal and tumour-bearing rats suggests that an important fraction of liposome-bound Hp is not complexed with serum albumin. It is conceivable that the gradual accumulation of Hp by tumour cells up to 72 h after administration of the porphyrin reflects a slow release of circulating Hp from the liposomes to the cell receptors. This hypothesis is supported by the experiments with liposomal HpdiMe. The latter porphyrin is endowed with a greater lipid-solubility than Hp, hence a consistently larger amount of porphyrin remains in the circulation and is found in the kidneys. Moreover, its accumulation by tumour cells occurs at a slower rate.

The detailed processes controlling the preferential uptake of porphyrins by tumour cells are still partially unknown. We are presently pursuing our studies with free and liposome-bound porphyrins to elucidate the detailed mechanism of their interactions with subcellular structures.

In any case, the prolonged retention of elevated amounts of liposomal Hp and HpdiMe opens the possibility of improving the efficacy of phototherapy whilst minimizing the onset of undesired side effects. Actually, preliminary experiments performed in our laboratory indicate a rapid regression of the above tumour if the rats are exposed to red light at approximately 72 h after i.p. injection of liposomal Hp or HpdiMe.

This work was supported by the Consiglio Nazionale delle Ricerche (Italy) under the Progetto Finalizzato "Controllo della crescita tumorale maligna", Contract No. 81.01314.96.

### References

BERENBAUM, M.C., BONNET, R. & SCOURIDES, P.A. (1982). *In vivo* biological activity of the components of hematoporphyrin derivative. *Br. J. Cancer,* **45,** 571.

DOUGHERTY, T.J., GRINDEY, G.B., FIEL, R., WEISHAUPT, K.R. & BOYLE, D.G. (1975). Photoradiation therapy. II. Cure of animal tumours with hematoporphyrin and light. *J. Natl Cancer Inst.,* **55,** 115.

DOUGHERTY, T.J., KAUFMAN, J.E., GOLDFARB, A., WEISHAUPT, K.R., BOYLE, D. & MITTLEMAN, A. (1978). Photoradiation therapy for the treatment of malignant tumours. *Cancer Res.,* **38,** 2628.

DOUGHERTY, T.J., LAWRENCE, G., KAUFMAN, J.E., BOYLE, D.G., WEISHAUPT, K.R. & GOLDFARB, A. (1979). Photoradiation in the treatment of recurrent breast carcinoma. *J. Natl Cancer Inst.,* **62,** 231.

FIGGE, F.H.J., WEILAND, G.S. & MANGANIELLO, L.O.J. (1948). Cancer detection and therapy. Affinity of neoplastic embryonic and traumatized regenerating tissues for porphyrins and metalloporphyrins. *Proc. Soc. Expt Biol. Med.,* **68,** 640.

FORBES, I.J., COWLED, P.A., LEONG, A.S.Y. & 4 others, (1980). Phototherapy of human tumours using hematoporphyrin derivative. *Med. J. Aust.,* **2,** 489.

GOMER, C.J. & DOUGHERTY, T.J. (1979). Determination of $^{3}H$-$^{14}C$ hematoporphyrin derivative distribution in malignant and normal tissue. *Cancer Res.,* **39,** 146.

GRANELLI, S.G., DIAMOND, I., McDONAGH, A.F., WILSON, C.B. & NIELSEN, S.L. (1975). Photochemotherapy of glioma cells by visible light and hematoporphyrin. *Cancer Res.,* **35,** 2567.

HAYATA, Y., KATO, H., KONAKA, C., ONO, J. & TAKIZAWA, N. (1982). Hematoporphyrin derivative and laser photoradiation in the treatment of lung cancer. *Chest,* **81,** 269.

JORI, G., PIZZI, G.B., REDDI, E. & 4 others, (1979). Time dependence of hematoporphyrin distribution in selected tissues of normal rats and in ascites hepatoma. *Tumori,* **65,** 425.

KESSEL, D. (1977). Effects of photoactivated porphyrins on cell surface of leukemia L1210 cells. *Biochemistry,* **16,** 3443.

MOAN, J., PETTERSEN, E.O. & CHRISTENSEN, T. (1978). The mechanism of photodynamic inactivation of human cells *in vitro* in the presence of hematoporphyrin. *Br. J. Cancer,* **39,** 398.

SATO, H. (1955). Intraperitoneal transplantation of the Yoshida ascites hepatoma to various American strains of rats. *J. Natl Cancer Inst.,* **15,** 1367.

TOMIO, L., REDDI, E., JORI, G., ZORAT, P.L., PIZZI, G.B. & CALZAVARA, F. (1980). Hematoporphyrin as a sensitizer in tumor phototherapy: effect of medium polarity on the photosensitizing efficiency and role of the administration pathway on the distribution in normal and tumor-bearing rats. In *Lasers in Photomedicine and Photobiology* (Eds. Pratesi & Sacchi), Berlin: Springer, p. 145.

TOMIO, L., ZORAT, P.L., JORI, G. & 4 others. (1982*a*). Elimination pathway of hematoporphyrin from normal and tumor-bearing rats. *Tumori,* **68,** 283.

TOMIO, L., ZORAT, P.L., CORTI, L. & 5 others, (1982*b*). Cancer phototherapy: Biochemical bases and experimental results. *Med. Biol. Envir.,* **10,** 301.

YOSHIDA, T. (1949). The Yoshida sarcoma, an ascites tumour. *Gann,* **40,** 1.